# Batch effect exerts a bigger influence on the rat urinary metabolome and gut microbiota than uraemia: a cautionary tale

**DOI:** 10.1186/s40168-019-0738-y

**Published:** 2019-09-02

**Authors:** David William Randall, Julius Kieswich, Jonathan Swann, Kieran McCafferty, Christoph Thiemermann, Michael Curtis, Lesley Hoyles, Muhammed Magdi Yaqoob

**Affiliations:** 1 Centre for Translational Medicine and Therapeutics, William Harvey Research Institute, Queen Mary University of London, Charterhouse Square, London, EC1M 8BQ UK; 2 Division of Integrative Systems Medicine and Digestive Diseases, Imperial College London, South Kensington Campus, London, SW7 2AZ UK; 3 Faculty of Dentistry, Oral and Craniofacial Sciences, King’s College London, Guy’s Hospital, Great Maze Pond, London, SE1 9RT UK; 4 School of Science and Technology, Nottingham Trent University, Clifton Lane, Nottingham, NG11 8NS UK

**Keywords:** Microbiome, Urinary metabolome, ^1^H-NMR spectroscopy, Uraemia, Batch effect, Hippurate, Lactate, Acetate

## Abstract

**Background:**

Rodent models are invaluable for studying biological processes in the context of whole organisms. The reproducibility of such research is based on an assumption of metabolic similarity between experimental animals, controlled for by breeding and housing strategies that minimise genetic and environmental variation. Here, we set out to demonstrate the effect of experimental uraemia on the rat urinary metabolome and gut microbiome but found instead that the effect of vendor shipment batch was larger in both areas than that of uraemia.

**Results:**

Twenty four Wistar rats obtained from the same commercial supplier in two separate shipment batches underwent either subtotal nephrectomy or sham procedures. All animals undergoing subtotal nephrectomy developed an expected uraemic phenotype. The urinary metabolome was studied using ^1^H-NMR spectroscopy and found to vary significantly between animals from different batches, with substantial differences in concentrations of a broad range of substances including lactate, acetate, glucose, amino acids, amines and benzoate derivatives. In animals from one batch, there was a complete absence of the microbiome-associated urinary metabolite hippurate, which was present in significant concentrations in animals from the other batch. These differences were so prominent that we would have drawn quite different conclusions about the effect of uraemia on urinary phenotype depending on which batch of animals we had used. Corresponding differences were seen in the gut microbiota between animals in different batches when assessed by the sequencing of 16S rRNA gene amplicons, with higher alpha diversity and different distributions of *Proteobacteria* subtaxa and short-chain fatty acid producing bacteria in the second batch compared to the first. Whilst we also demonstrated differences in both the urinary metabolome and gut microbiota associated with uraemia, these effects were smaller in size than those associated with shipment batch.

**Conclusions:**

These results challenge the assumption that experimental animals obtained from the same supplier are metabolically comparable, and provide metabolomic evidence that batch-to-batch variations in the microbiome of experimental animals are significant confounders in an experimental study. We discuss strategies for reducing such variability and the need for transparency in research publications about the supply of experimental animals.

**Electronic supplementary material:**

The online version of this article (10.1186/s40168-019-0738-y) contains supplementary material, which is available to authorized users.

## Background

The lack of reproducibility in pre-clinical animal research remains a major challenge in experimental biology [1] and is at least partially explained by variation between animal microbiomes [2]. Animal research has been based on the assumption that whilst experimental animals in different facilities may have differences at species level between their gut microbiota [3], at a population level, in healthy laboratory animals on identical diets, these diverse collections of microorganisms achieve a shared set of basic metabolic functions—an assumption supported by evidence of significant functional redundancy within gut microbial communities [4].

A number of toxic molecules that accumulate in renal failure have been shown to be produced by bacterial metabolism of dietary protein in the large intestine [5, 6], leading to an interest in the gut microbiome as a potential therapeutic target to reduce the cardiovascular morbidity of patients with chronic kidney disease [7].

Based on an assumption of metabolic similarity between experimental animals, we sought to investigate this ‘gut-kidney axis’ in a rodent model of uraemia, by demonstrating the effect of experimental uraemia on the urinary metabolome and gut microbiota of rats, purchased from the same supplier in two separate shipment batches for logistical reasons. We actually found that the effect of shipment batch had a larger effect in both areas than uraemia and that conclusions drawn about the effect of uraemia on gut-derived metabolites would have been radically different depending on the batch of animals used.

## Results

**Fig. 1:**
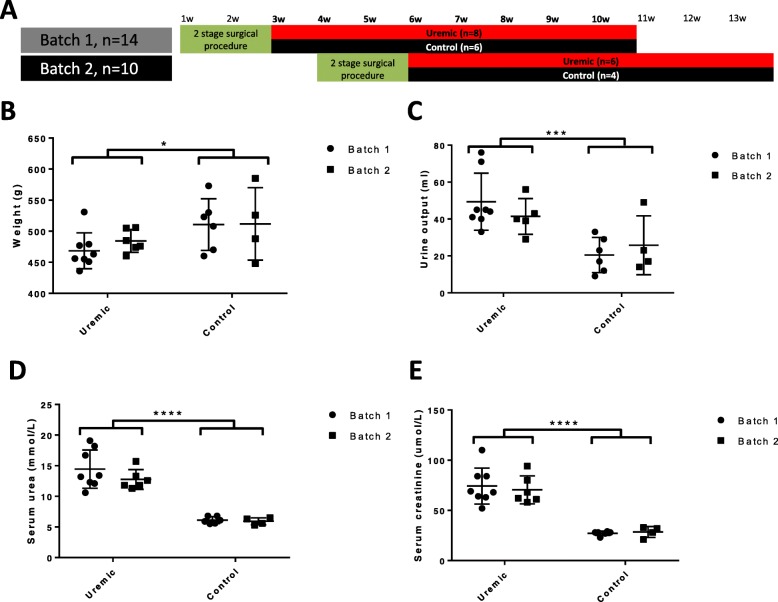
Animal work. **a** Outline of experimental procedures. Time in weeks is shown along the top of the figure. Animals arrived in two batches, 3 weeks apart, at age 7 weeks, and after a week-long acclimatisation period, underwent a 2-stage subtotal nephrectomy or sham procedure. Eight weeks after the second stage of this procedure, after a 24-h urine collection, they were sacrificed and samples of serum and caecal fluid collected. **b** Weight at time of sacrifice (*p* = 0.033 for treatment, *p* = 0.586 for batch, by 2-way ANOVA). **c** 24 h urine volumes immediately before sacrifice (*p* = 0.0009 for treatment, *p* = 0.256 for batch, by 2-way ANOVA). **d** Serum urea at time of sacrifice (*p* < 0.0001 for treatment, *p* = 0.392 for batch, by 2-way ANOVA). **e** Serum creatinine at the time of sacrifice (*p* < 0.0001 for treatment, *p* = 0.645 for batch, by 2-way ANOVA)

We obtained 24 wild-type outbred Wistar International Genetic Standard (IGS) rats in two shipment batches, 3 weeks apart, from the same supplier (Charles Rivers, Kent, UK). Fourteen were rendered uraemic by undergoing a two-stage subtotal (five-sixth) nephrectomy (eight from batch 1, six from batch 2), whilst 10 underwent sham procedures (six from batch 1, four from batch 2, Fig. 1a). There were no differences in animal husbandry or diet between batches. At the time of sacrifice 8 weeks later, the urinary metabolome was assessed by untargeted proton nuclear magnetic resonance (^1^H-NMR) spectroscopy, and composition of the gut microbiota was assessed by sequencing 16S rRNA gene amplicons. All animals undergoing subtotal nephrectomy developed an expected uraemic phenotype, including elevations in serum urea and creatinine, weight loss, and polyuria compared to sham-operated controls, and there were no gross phenotypic differences between animals from different batches (Fig. 1b–f; Additional file 1).

**Fig. 2:**
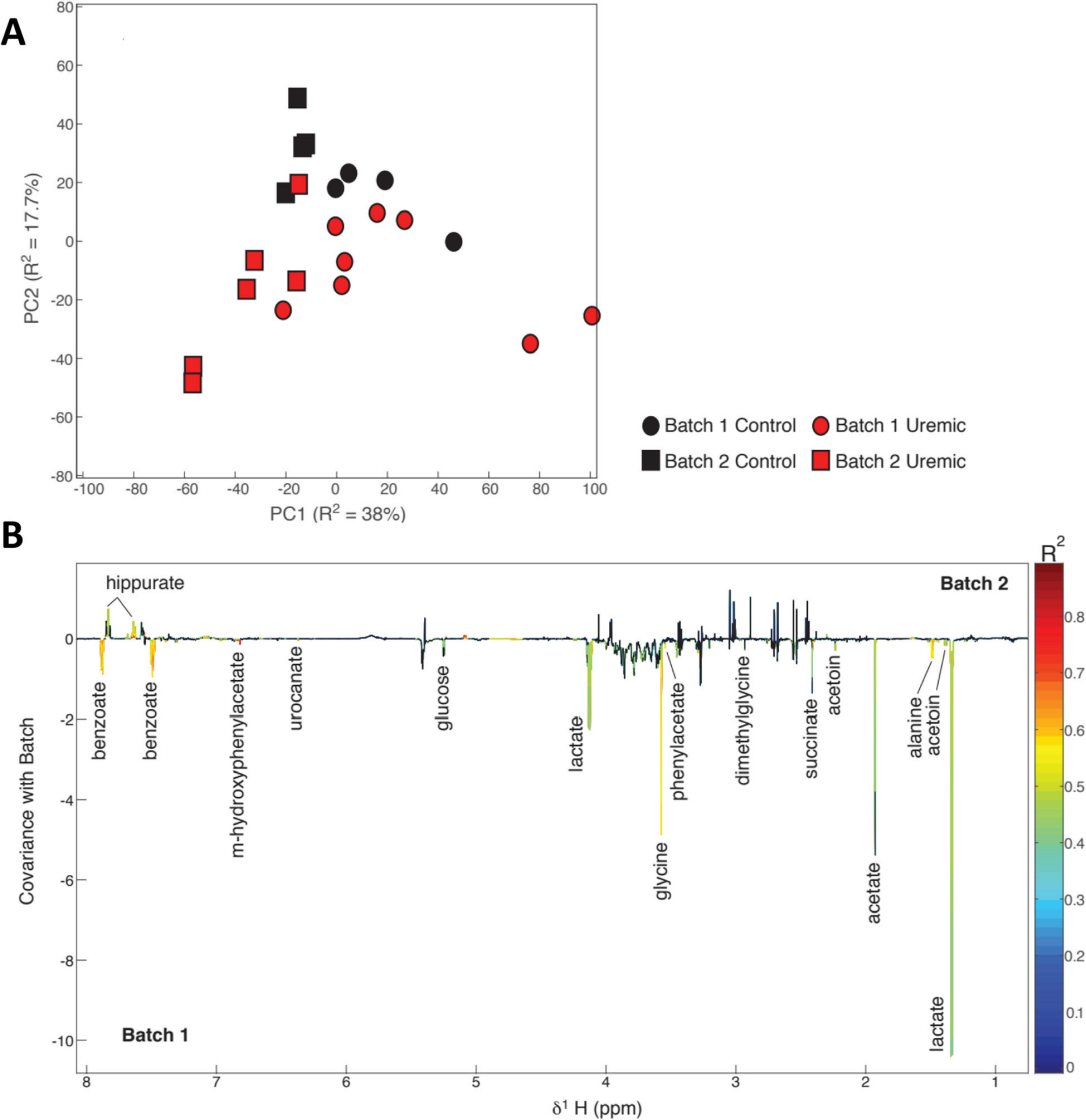
Untargeted ^1^H-NMR spectroscopy of 24-h rat urine collections. **a** Score plot of unsupervised principal component analysis of normalised and aligned NMR spectra, showing that samples separated when analysed by batch chiefly in the first principal component, which accounted for 38% of total variance, and separated when analysed by surgical treatment chiefly in the second principal component, which accounted for 17.7% of variance. **b** Loading plot from an orthogonal projection to latent squares discriminant analysis (OPLS-DA) model built using shipment batch as the response variable, back-plotted as an NMR spectrum with peak height indicating covariance with batch (downwards deflections indicate substances more abundant in animal urine from batch 1; upwards deflections indicate substances more abundant in animal urine from batch 2). The line is coloured according to the significance of the association, adjusted for multiple testing using the Benjamini-Hochberg method; black indicates non-significance between groups. Peaks are labelled with the identity of the responsible substance

Principal component analysis (PCA) of normalised and aligned urinary NMR spectral profiles identified that shipment batch was responsible for the largest source of variance in the biochemical data, seen chiefly in principal component 1, which accounted for 38% of variance. Surgical treatment accounted for a smaller but nonetheless definite source of variance, with these differences being seen chiefly in the second principal component, which accounted for 17.7% of total variance (Fig. 2a).

Separate orthogonal projection to latent structures discriminant analysis (OPLS-DA) models was constructed to elucidate biochemical variation associated with shipment batch and treatment class. The model built using shipment batch had a stronger predictive power (*Q*^2^*Y* = 0.66, *p* = 0.001) than the model built using treatment class (*Q*^2^*Y* = 0.48, *p* = 0.007). Discriminatory metabolites between the two shipment batches were identified from the OPLS-DA model (Fig. 2b), and their relative abundances were calculated from integration of the relevant regions of the aligned spectral profiles (Table 1).

**Table 1:** Normalised relative concentrations of selected urinary metabolites (relative units)

Substance	Batch 1	Batch 2	*p* ^ǂ^	Uraemic	Control	*p* ^ǂ^
Acetamide	28.803	28.126	0.930	24.867	34.845	*0.001*
Acetate	192.187	105.217	*0.010*	160.957	138.128	0.776
Acetoin	9.593	8.767	0.192	8.957	9.674	0.188
Alanine	29.330	18.013	*0.001*	23.765	24.923	0.809
Allantoin	28.996	29.158	0.967	25.391	35.508	0.054
Benzoate	110.964	52.071	*< 0.001*	82.269	87.564	0.809
Betaine	55.595	39.399	0.129	47.318	49.834	0.809
Citrate	119.823	112.407	0.752	126.188	99.414	0.127
Creatinine	140.283	152.104	0.642	131.066	171.189	*0.027*
Dimethylamine	21.667	21.548	0.967	19.390	25.504	*0.054*
Dimethylglycine	15.669	12.643	0.124	14.725	13.538	0.677
Formate	2.873	3.007	0.967	1.995	4.575	0.127
Glucose	43.856	19.678	*0.018*	34.385	30.208	0.809
Glycine	141.491	68.457	*< 0.001*	105.888	112.505	0.809
Hippurate	6.559	34.509	*0.010*	14.556	27.501	0.533
Lactate	571.659	188.265	*0.005*	402.362	388.686	0.922
m-Hydroxyphenylacetate	7.086	5.944	0.827	5.387	8.632	0.600
2-Oxoglutarate	167.931	182.841	0.642	183.945	158.543	0.533
Phenylacetate	13.308	8.148	*0.001*	10.380	11.982	0.600
Pyruvate	5.064	6.344	0.659	4.855	7.028	0.600
Succinate	97.877	72.642	*0.044*	85.106	88.682	0.809
Taurine	37.782	29.758	0.573	23.957	51.946	*0.009*
Trimethylamine	5.214	16.793	0.124	13.864	4.549	0.159
Trimethylamine *N*-oxide	42.391	32.387	0.253	34.013	44.547	0.267
Trigonelline	− 0.013	− 0.024	0.218	− 0.023	− 0.009	0.059
Urocanate	2.764	1.001	*< 0.001*	2.056	1.799	0.776

^ǂ^*p* values calculated using Student’s *t* test with Welch’s correction for unequal variances, subsequently adjusted to limit the false discovery rate to 0.15 using the Benjamini-Hochberg procedure [8]. Values in italic are significant at this level

Animals in batch 1 excreted significantly greater amounts of glycine (141.5 vs 68.5 relative units, Benjamini-Hochberg adjusted *p* < 0.001), alanine (29.3 vs 18.0 units, *p* < 0.001) and glucose (43.9 vs 19.7 units, *p* = 0.006) than animals in batch 2. They also excreted higher amounts of the potential gut bacterial products acetate (a short-chain fatty acid, 192.2 vs 105.2 units, *p* = 0.003), succinate (a bacterial metabolic product of dietary fibre digestion, 97.9 vs 72.6 units, *p* = 0.017) and lactate (571.7 vs 188.3 units, *p* = 0.001), compared with those in batch 2. Interestingly, hippurate was almost completely absent from the urine of batch 1 animals but present in urine from all animals in batch 2 (6.6 vs 34.5 units, *p* = 0.003). Correspondingly, benzoate, a gut microbially derived precursor of hippurate, was lower in the urine of batch 2 animals compared to those in batch 1 (111.0 vs 52.1 units, *p* < 0.001). Whilst a high degree of between-sample variation meant the batch effect did not reach the overall significance, on review of individual sample NMR spectra, it became clear that many animals had no detectable trimethylamine (TMA), a product of bacterial protein metabolism, including almost all of those in batch 1, whereas others (predominantly those in batch 2) had easily detectable concentrations.

To determine whether the substantial batch variations we had demonstrated could have led to erroneous conclusions about the effect of uraemia on the urinary metabolome, we built an OPLS-DA model for each shipment batch separately using surgical treatment class (subtotal nephrectomy vs sham) as the response variable. The model built on the batch 1 profiles was not found to be significant (*Q*^2^*Y* = 0.265, p*Q*^2^*Y* = 0.120), leading to the potential conclusion that the urinary metabolome is not influenced by uraemia. However, a significant predictive model was obtained using profiles from batch 2 (*Q*^2^*Y* = 0.543, p*Q*^2^*Y* = 0.049), despite small sample numbers, suggesting that uraemia does indeed determine urinary phenotype.

To assess whether differences in the gut microbiota between shipment batches and treatment classes might underlie these trends in the metabolomic data, sequencing of the V3 and V4 hypervariable regions of the amplified 16S rRNA gene in DNA extracted from caecal fluid was carried out. Sequence abundance data underwent isometric log-ratio transformation to allow compositional analysis of the different microbial communities.

**Fig. 3:**
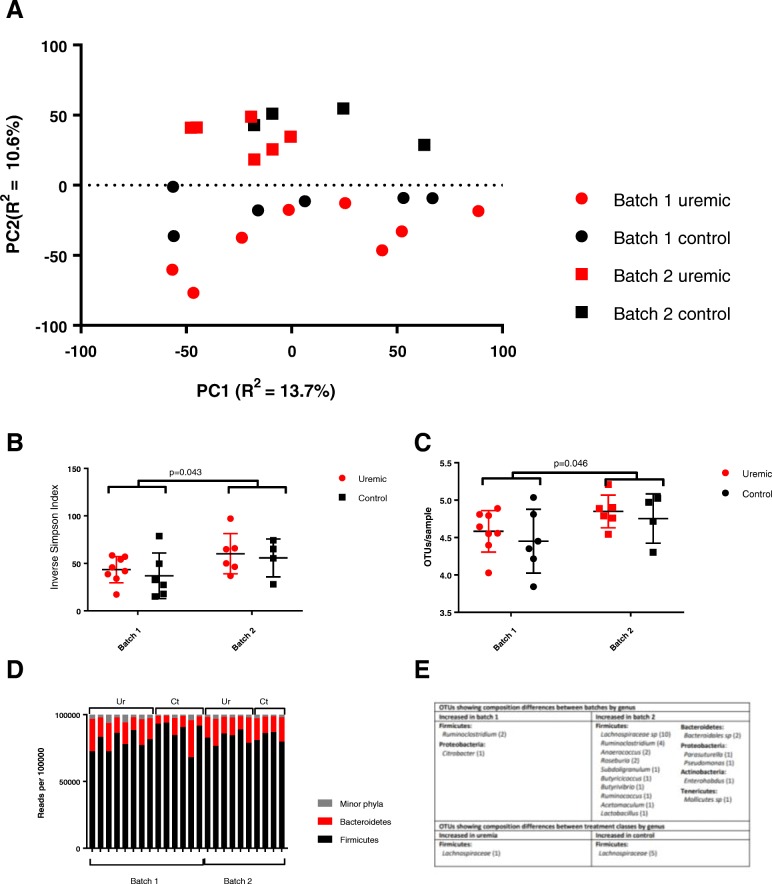
Next-generation sequencing of the 16S rRNA gene amplicon from caecal fluid. **a** Untargeted principal component analysis of log-ratio transformed OTU abundance by sample, showing closer clustering associated with shipment batch than with treatment class. **b**, **c** Alpha diversity, measured by the inverse Simpson index (40.7 vs 58.5, *p* = 0.043 by Student’s *t* test with Welch’s correction, **b**) and Shannon index (4.53 vs 4.81, *p* = 0.046, **c**). **d** Relative abundances of major phyla in each sample, grouped by batch and treatment group. There were no significant differences when analysed by batch or by treatment groups. **e** Taxonomic attributions of OTUs differentially abundant when analysed by shipment batch and treatment class, assessed using the Analysis of Composition of Microbiomes (ANCOM) framework with alpha set at 0.05 and a cutoff value of 0.6

Unsupervised PCA of the compositional data revealed that shipment batch had a larger impact on sample clustering than did treatment class (Fig. 3a). Consistent with this, permutational multivariate analysis of variance (PERMANOVA) was performed using an ADONIS analysis of a Euclidean distance matrix and confirmed that batch had a small but significant effect on the gut microbiome (*R*^2^ = 0.097, *p* = 0.001), whilst treatment class did not (*R*^2^ = 0.048, *p* = 0.227). This was further confirmed by showing that a valid predictive OPLS-DA model could be built using shipment batch as the response variable (*Q*^2^*Y* = 0.573, *p* < 0.05), but not when using treatment class (*Q*^2^*Y* = 0.206, *p* = 0.2).

The gut microbiotas of animals differed significantly in community structure between batches, with samples taken from animals in batch 2 displaying higher alpha diversity than those from animals in batch 1, across a range of measures including the inverse Simpson (40.7 vs 58.5, *p* = 0.043, Fig. 3b) and Shannon indices (4.53 vs 4.81, *p* = 0.046, Fig. 3c). Conversely, we did not demonstrate a difference in alpha diversity between uraemic and control animals.

To explore these differences more closely, populations were assessed on the basis of taxonomic assignments of OTUs at phylum, order, class, family and genus levels. Microbiotas in all animals were dominated by phyla *Firmicutes* (accounting for 83.1% of total reads) and *Bacteroidetes* (14.5%), with all other phyla (*Verrucomicrobia*, *Tenericutes*, *Proteobacteria*, *Actinobacteria*, *Saccharibacteria* and *Deferribacteres*) together representing less than 2.5% of total sequences when normalised across samples (Fig. 3d).

Differences in the abundances of OTUs and higher taxonomic groupings were analysed between shipment batches and treatment classes using the Analysis of Composition of Microbiomes (ANCOM) framework, based on isometrically log-ratio transformed abundance data and Benjamini-Hochberg adjustment for multiple hypothesis testing. Differential abundances between samples taken from animals in different shipment batches were apparent as high as at class level, with animals in batch 2 having higher relative abundances of *Pseudomonadales* in phylum *Proteobacteria*. No higher-order differences were demonstrated between uraemic and control animals.

On further analysis at OTU level, it became clear that it was primarily the less abundant OTUs which showed significant differences between batches, whilst OTUs differing significantly between uraemic and control animals were generally more abundant. Thus, whilst the relative abundance of 33/1110 OTUs (2.97% on the total) differed significantly between shipment batches, these represented only 3.80% of total sequences when analysed by the abundance of each OTU. However, the six OTUs which differed significantly between treatment classes (0.54% of the total) accounted for 5.13% of total sequences when adjusted for abundance.

These six OTUs showing significant abundance differences between uraemic and control animals were all from the family *Lachnospiraceae*: five from the NK4A136 group and one from the UCG-001 group. All but one showed significant decreases in relative abundance in uraemic animals, including the third most abundant OTU overall.

The 33 OTUs showing significant compositional differences between batches were drawn from five different phyla. In keeping with the higher alpha diversity seen in samples from batch 2 animals, 30/33 differentially abundant OTU between batches were seen in higher abundances in animals from this batch. Interestingly bacterial genera known to possess significant metabolic potential were prominently represented amongst these differentially abundant organisms, including a number of producers of short-chain fatty acids (*Roseburia*, *Butyricicoccus*, *Butyrivibrio* and *Acetomaculum*) and three from the phylum *Proteobacteria.*

## Discussion

The rodent gut microbiome is a complex community of several hundred different bacterial species that possess significant metabolic potential of immense relevance to the host organism. It has previously been demonstrated that this community differs according to a variety of factors including host age [9] and genetics [10, 11], caging arrangements [9, 11, 12], bedding material and water sterilisation technique [13] and vendor shipment batch [10]. Xiao et al. generated a catalogue of the mouse metagenome by sequencing faecal material from 184 mice and found that vendor was a prime determinant in variation at a genetic and function level [14].

In light of these studies, we have demonstrated that predicted batch variations in gut microbiota are associated with multiple, major variations in a range of urinary metabolites, with the potential for significant downstream effects on wider areas of host phenotype. For example, circulating hippurate has recently been suggested as a biomarker for gut microbial diversity, associating with the risk of metabolic syndrome [15]; however, our results suggest it may be totally absent in the urine of experimental animals based on shipment batch. Likewise, the biological relevance of dietary amines has been demonstrated through the association of TMA and its metabolite trimethylamine *N*-oxide with cardiovascular disease [16, 17], including in patients with chronic kidney disease [18]. However, our results suggest that rats purchased from the same supplier in different shipment batches may metabolise dietary amines in quite different ways, potentially questioning the generalisability of research based on individual batches of animal subjects.

Since the diet of animals in each group was identical, we conclude that differences in bacterial metabolic pathways are likely to underlie these differences in the urinary metabolome. We demonstrated batch differences in the relative abundances of a number of bacteria that are of known metabolic significance, including several that are major sources of short-chain fatty acids and associated with beneficial health outcomes [19–21], and several from the phylum *Proteobacteria* that has recently been shown to contribute significantly to functional variation between gut metagenomes [22].

These results challenge the assumption that in healthy organisms, different microbial communities achieve a common set of basic metabolic functions despite variation in the individual species present [23, 24]. It can no longer be assumed that healthy laboratory animals, purchased from the same supplier, are metabolically similar. The inherent microbial dissimilarity and associated metabolic differences between animals in different batches provide a significant source of experimental variation.

Such batch variations could easily lead to spurious positive results. For example, a group that demonstrates an effect in response to an experimental intervention with a small group of animals may decide to increase the number of animals in order to publish their findings; they purchase new animals from the same supplier, but fail to reproduce their earlier results because the new additions have significantly different microbial metabolic potential. Even worse, they may have carried out interventional procedures on one batch of animals, and then used animals from a different batch as controls, with exaggerated differences between groups reflecting underlying differences in microbiomes rather than any effect of the experimental procedure. The alternative in each case—to re-run the whole experiment with animals purchased in a new, single batch—may be prohibitively expensive, may fail to reproduce the initial results and seems to stand against the second of the ‘Three R’s’ governing ethical use of animals in research: the reduction of the number of animals used [25].

## Conclusions

It is crucial that publishers maintain the requirement to document fully all aspects of animal use, including purchase details of the different batches of animals used in a study if these come from a commercial supplier. Furthermore, steps should be taken to reduce the amount of variation within batches, such as by using a standardised procedure for moving bedding between cages, which has been shown to reduce intra-batch variation [26]. Many experimental groups breed their own animals, which may reduce intra-group variation, although potentially at the expense of generalisability with results from other laboratories. Statistical approaches including percentile normalisation have been suggested that would allow pooling of data between different batches on experimental subjects in different settings, although for this to be successful, large numbers of control subjects are required [27].

Finally, batch variation can be embraced as a reflection of real-world microbial variation. For this to be successful, it is important that researchers use unsupervised PCA plots of all experimental subjects, coloured according to batch, for quality control, using statistical methods to measure the effect of batch variation. Documenting whether the same observed changes were seen in animals from all batches, or whether different batches behaved differently, is helpful in assessing the generalisability of results, and many journals already have such requirements in place. An experiment showing the same effect in two or more smaller but separate batches of animals may be more striking than an experiment showing a larger effect size in a single animal batch.

## Methods

### Animal work

Animal experiments were conducted in accordance with the UK Home Office Animals (Scientific Procedures) Act 1986, with local ethical committee approval. All animal works were carried out at the Biological Services Unit of Queen Mary University of London at Charterhouse Square, and complied fully with all relevant animal welfare guidance and legislation. The 24 male, outbred Wistar IGS rats were obtained from Charles Rivers (Kent, UK) in two shipment batches 3 weeks apart. All were housed in individually ventilated cages under 12-h light/dark cycles and were allowed unlimited access to water and chow (RM1 diet from Special Diet Services, Essex, UK).

After a week-long period of acclimatisation, rats underwent a two-stage surgical procedure involving either subtotal nephrectomy or a sham procedure. Subtotal nephrectomy involved exteriorisation of the left kidney with decapsulation and removal of the upper and lower poles and subsequent replacement of the middle pole only, followed by total right nephrectomy 2 weeks later. Sham procedures involved exteriorisation, decapsulation and replacement of the left kidney, followed by the same procedure on the right kidney 2 weeks later.

Following surgery, rats were weighed weekly. There were up to four rats per cage, and the animals were initially housed according to surgical intervention (subtotal nephrectomy or sham) for 2 weeks after the second stage surgery, before some were moved into mixed cages comprising both uraemic and control animals (this was in order to assess the role of individual cage variants, which on subsequent analysis, not presented here, was found not to be as important in explaining the key changes in urinary metabolome or gut microbiome as shipment batch or treatment class). There was no co-housing between batches. Each week, the animals were housed individually in metabolism cages to allow the collection of a 24-h urinary specimen which was frozen at − 80 °C until the time of analysis. Rats were killed by lethal injection of sodium thiopentone (LINK Pharmaceuticals, Horsham, UK), and caecal fluid was expressed, stored in foil and snap-frozen in liquid nitrogen and then at − 80 °C until the time of analysis. Blood samples were taken by cardiac puncture, and after centrifugation, the serum was frozen at − 80 °C until the time of analysis.

### Plasma biochemistry

Quantification of serum urea and creatinine was done by IDEXX Bioresearch, Ludwigsberg, Germany.

### NMR spectroscopy

Urine samples were randomised prior to dilution with buffer and running on the machine to remove potential for technical batch effects in processing and analysis, and prepared for ^1^H-NMR spectroscopy as described previously [28]. All samples were analysed on an NMR spectrometer (Bruker) operating at 600.22 MHz ^1^H frequency.

### Processing of NMR data

The NMR spectral profiles were digitised and imported into Matlab (Mathworks) using in-house scripts (Additional file 3). The raw spectra were adjusted for 24-h urine volumes by multiplying all NMR absorbance values by the urine volume in millilitres. The peaks for water and trimethylsilylpropanoic acid (TSP) were excised from the raw NMR spectra which were then aligned to adjust for variation in peak shift due to pH differences. Further normalisation was carried out using the probabilistic quotient method between samples in order to ensure comparable baselines between samples (Additional file 2).

Unsupervised PCA was used to identify sources of variation in the metabolic data. This was followed by supervised OPLS-DA analysis using both shipment batch and treatment class as the response variable. In-house-developed scripts were used to perform these multivariate statistical analyses. Peak integrals were calculated from metabolite peaks identified as discriminatory from the OPLS-DA models. Comparisons between these integrals were used to calculate differences in relative abundance according to shipment batch and treatment class using Microsoft Excel, with the Student’s *t* test and Welch’s correction used to assess significance. These *p* values were adjusted using the Benjamini-Hochberg method [8] and a false discovery rate of 0.15 using the *q* values [29] package in R (Additional file 4).

### 16S rRNA gene sequencing and analyses

DNA was extracted from samples of caecal fluid using the DNeasy PowerSoil kit from QIAGEN, used according to the manufacturer’s instructions. All samples were processed using the same kit, and a negative ‘kitome’ control was also included with samples [30]. DNA diluted to 10 ng/μL (in 10 mM Tris HCl pH 8.5) was submitted to the Centre for Genomic Research at the University of Liverpool for library preparation and sequencing of the V3/V4 hypervariable region of the 16S rRNA gene. Sequence data were processed using QIIME v1.9 [31]. Paired-end data were joined using join_paired_ends.py, and primer sequences removed from split library files using cutadapt [32]. OTUs were picked using 99% BLAST identity using usearch; from these, a representative set of OTUs was selected. Sequences were aligned (PyNAST) against Silva v128 [33], and this database was also used to assign taxonomy. Singletons, mitochondria-, cyanobacteria- and control-associated OTUs were removed from the OTU table, as were OTUs unaffiliated with any taxonomic group. Data were then rarefied to 100,000 reads to account for differences in sequencing depth across samples and these relative abundances were used to calculate the overall abundances by phylum presented in Fig. 3d.

Raw (unrarefied) OTU abundance data were imported into R for analyses using *Phyloseq* [34] (Additional files 5 and 6). A phylogenetic tree was generated using MEGA v7.0 [35] and rooted to a random node using the R package *phytools* [36]. A pseudocount of 0.001 was added to all OTU abundances to avoid calculating log-ratios involving zeros, and then data was then made compositional through isometric log-ratio transformation using the R package *philr* [37]. Ordination was carried out using the ‘ordinate’ function in *Phyloseq*, based on Euclidean distances in philr space. Permutational analysis of variance (PERMANOVA) was carried out using the ADONIS command in the R package *vegan* [38]. OPLS-DA models were built using the *ropls* package in R [39]. Alpha diversity was assessed using *Phyloseq.* Compositional analysis of the microbiota at six taxonomic levels was based on isometric log-ratio transformation of raw sequence abundances and adjusted for multiple testing using the Benjamini-Hochberg method, carried out using the *ANCOM* statistical framework [40] in R, with code obtained from the author’s webpage: https://sites.google.com/site/siddharthamandal1985/research.

### Preparation of figures

In order to achieve uniformity, most figures except those demonstrating NMR spectral data were generated using GraphPad Prism 7 (GraphPad Software Inc., San Diego, California). NMR spectra and related figures were created using Matlab (Mathworks) with in-house scripts.

## Additional files

Additional file 1: Animal data (XLSX 11 kb)

Additional file 2: Raw NMR data (CSV 3674 kb)

Additional file 3: MATLAB code for NMR analysis (DOCX 17 kb)

Additional file 4: R code for NMR analysis (DOCX 17 kb)

Additional file 5: Non-rarefied 16S abundance data (CSV 77 kb)

Additional file 6: R code for microbiome analysis (DOCX 25 kb)

## Data Availability

Animal data (weight, urine output and serum biochemistry) are submitted as Additional file 1. The raw NMR data is available as Additional file 2. The rarefied 16S rRNA gene sequence data including the negative ‘kitome’ control have been deposited with links to BioProject accession number PRJNA525754 in the NCBI BioProject database (https://www.ncbi.nlm.nih.gov/bioproject/). Raw (non-rarefied) data are available as Additional file 5.

## Abbreviations

IGS: International Genetic Standard
NMR: Nuclear magnetic resonance
OPLS-DA: Orthogonal projection to latent structures discriminant analysis
OTU: Operational taxonomic unit
PCA: Principal component analysis
TMA: Trimethylamine
TSP: Trimethylsilylpropanoic acid
