# Supplementary material for: Batch effect exerts a bigger influence on the rat urinary metabolome and gut microbiota than uraemia: a cautionary tale
Source: Microbiome. 2019 Sep 2;7:127. doi: 10.1186/s40168-019-0738-y (PMC6720068; doi:10.1186/s40168-019-0738-y)
Supplement: Supplementary file 3 — MATLAB code for NMR analysis (DOCX 17 kb) [file 40168_2019_738_MOESM3_ESM.docx]

**Batch effect exerts a bigger influence on the rat urinary metabolome and gut microbiota than uremia: a cautionary tale**

Randall D.W. *et al.*

This document contains the statistical analyses performed on the metabolome data for the manuscript. All data to reproduce analysis can be found online at Metabolights (<https://www.ebi.ac.uk/metabolights/>) using the accession number MTBLS1010.

Note that user-specific variation will occur when manually aligning the NMR spectral data. As a result, slight variation will occur during reanalysis of raw NMR spectral data, though core results will remain essentially unchanged.

These steps are to be performed on the imported and processed spectra data (nmr_spectra). Class information should be provided in the vector ‘classes’ (Batch 1 = 1 and Batch 2 = 2).

**Protocol**

1. **Install IMPaCTS toolbox**

(online documentation: <https://csmsoftware.github.io/docs/impacts/getting-started.html>)

- 1. Navigate to: <https://github.com/csmsoftware/IMPaCTS>
  2. Clone or download the repo found on github
  3. Add the IMPaCTs Toolbox to Matlab search path

1. **Perform principal components analysis (PCA)**

*Build a PCA model with 2 principal components using pareto scaling*

>> PCA = csm_pca (nmr_spectra, 2, 'prep', 'pa');

*Plot scores plot*

>> figure = csm_plot_pca (PCA, 'plot_type', 'scores', 'classes', classes);

1. **Perform orthogonal projection to latent structures-discriminant analysis (OPLS-DA)**

*Build an OPLS-DA model with 1 predictive component and 2 orthogonal components*

>> OPLSDA = csm_orth_pls (csm_spectra, classes, 'num_pred_comp', 1, 'num_Y_orth_comp', 2, 'num_cv_rounds',7, 'scale_type', 'mc', 'model_type', 'da');

*Output Q^2^Y value (predictive ability) of model*

>> OPLSDA.output.cv.Q2Yhat

ans =

0.4040

0.5074

0.6606

*Perform permutation testing (1000 permutations) to compare the OPLS-DA model against a randomized version to test the validity.*

>> OPLSDA_perm = csm_orth_pls_permutate (csm_spectra, classes, 1000, 'num_pred_comp', 1, 'num_Y_orth_comp', 2, 'num_cv_rounds', 7, 'scale_type', 'mc', 'model_type', 'da');

>> OPLSDA_perm.output.pv

ans =

1.0000e-03

*Plot the coefficients plot from the OPLS-DA model*

>> figure = csm_plot_orth_pls(OPLSDA);
