# Supplementary material for: Batch effect exerts a bigger influence on the rat urinary metabolome and gut microbiota than uraemia: a cautionary tale
Source: Microbiome. 2019 Sep 2;7:127. doi: 10.1186/s40168-019-0738-y (PMC6720068; doi:10.1186/s40168-019-0738-y)
Supplement: Supplementary file 4 — R code for NMR analysis (DOCX 17 kb) [file 40168_2019_738_MOESM4_ESM.docx]

**R scripts used in NMR data analysis:**

R version 3.6.0 (2019-04-26) -- "Planting of a Tree"

Copyright (C) 2019 The R Foundation for Statistical Computing

Platform: x86_64-w64-mingw32/x64 (64-bit)

#Load required packages

> library(qvalues)

#Import p values, generated using TTEST function in Microsoft excel (Student t-test with Welch’s correction for unequal variances), based on MATLAB integrated peak height data, according to either shipment batch or treatment class:

> batch <- read.csv(filePath)

> batch

Substance p

Acetamide 8.224072e-01

Acetate 2.610866e-03

Acetoin 1.034856e-01

Alanine 8.477800e-05

Allantoin 9.669511e-01

Benzoate 1.862870e-05

Betaine 6.433772e-02

Citrate 6.075106e-01

Creatinine 4.668713e-01

Dimethylamine 9.654235e-01

Dimethylglycine 5.727902e-02

Formate 9.083113e-01

Glucose 6.095045e-03

Glycine 4.712510e-06

Hippurate 3.160813e-03

Lactate 1.103678e-03

m-hydroxyphenylacetate 6.997779e-01

2-oxoglutarate 4.694820e-01

Phenylacetate 2.428720e-04

Pyruvate 5.066873e-01

Succinate 1.705074e-02

Taurine 3.747647e-01

Trimethylamine 5.500364e-02

Trimethylamine-N-oxide 1.557699e-01

Trigonelline 1.258852e-01

Urocanate 4.450910e-05

> treatment <- read.csv(filePath)

> treatment

Substance p

Acetamide 0.0000208806

Acetate 0.5543651420

Acetoin 0.0723686360

Alanine 0.7660883810

Allantoin 0.0104001410

Benzoate 0.7729471030

Betaine 0.7777481700

Citrate 0.0390092190

Creatinine 0.0031339800

Dimethylamine 0.0085503690

Dimethylglycine 0.4425348420

Formate 0.0362594600

Glucose 0.6255979460

Glycine 0.7610684160

Hippurate 0.2667464540

Lactate 0.9221976440

m-hydroxyphenylacetate 0.3564878530

2-oxoglutarate 0.2473713390

Phenylacetate 0.3632427400

Pyruvate 0.3692804240

Succinate 0.7660090820

Taurine 0.0006688600

Trimethylamine 0.0549944300

Trimethylamine-N-oxide 0.1131050090

Trigonelline 0.0135116960

Urocanate 0.5672437800

#Extract p values

> pbatch <- batch$p

> ptreatment <- treatment$p

#Generate q value object (qvalue_truncp function used for the treatment class analysis as one of the p values was extremely small)

> qobjbatch <- qvalue(p = pbatch)

> qobjtreatment <- qvalue_truncp(p = ptreatment)

#Extract q values

> qbatchvalues <- qobjbatch$qvalues

> qbatchvalues

0.9296776829

0.0096975023

0.1921874927

0.0005510570

0.9669511190

0.0002421731

0.1286754300

0.7521559413

0.6424491046

0.9669511190

0.1241045347

0.9669511190

0.0176079078

0.0001225253

0.0102726423

0.0047826047

0.8270102785

0.6424491046

0.0012629344

0.6586934783

0.0443319136

0.5731694724

0.1241045347

0.2531261021

0.2182010185

0.0003857455

> qtreatmentvalues <- qobjtreatment$qvalues

> qtreatmentvalues

0.000376097

0.537740864

0.130348875

0.560345500

0.037465033

0.560345500

0.560345500

0.087828237

0.018816198

0.037465033

0.468873240

0.087828237

0.560345500

0.560345500

0.369583183

0.638862739

0.415712614

0.369583183

0.415712614

0.415712614

0.560345500

0.006023683

0.110060909

0.185202162

0.040561641

0.537740864
