# Supplementary material for: Batch effect exerts a bigger influence on the rat urinary metabolome and gut microbiota than uraemia: a cautionary tale
Source: Microbiome. 2019 Sep 2;7:127. doi: 10.1186/s40168-019-0738-y (PMC6720068; doi:10.1186/s40168-019-0738-y)
Supplement: Supplementary file 6 — R code for microbiome analysis (DOCX 25 kb) [file 40168_2019_738_MOESM6_ESM.docx]

**R scripts used in microbiome analysis:**

R version 3.6.0 (2019-04-26) -- "Planting of a Tree"

Copyright (C) 2019 The R Foundation for Statistical Computing

Platform: x86_64-w64-mingw32/x64 (64-bit)

#Load required packages

> library(phyloseq)

> library(vegan)

> library(ropls)

> library(philr)

> library(phytools)

> library(exactRankTests)

> library(nlme)

#Import data, previously saved as phyloseq object

> snx<-readRDS(filePath)

> snx

phyloseq-class experiment-level object

otu_table() OTU Table: [ 1110 taxa and 24 samples ]

sample_data() Sample Data: [ 24 samples by 3 sample variables ]

tax_table() Taxonomy Table: [ 1110 taxa by 8 taxonomic ranks ]

phy_tree() Phylogenetic Tree: [ 1110 tips and 1109 internal nodes ]

#Label nodes, then choose random OTU and root tree

> phy_tree(snx <- makeNodeLabel(phy_tree(snx), method=”number”, prefix=’n’)

> rand <-sample(1:1110,1)

> rand

1019

> phy_tree(snx) <- reroot(phy_tree(snx), 1019)

>phy_tree(snx)

Phylogenetic tree with 1110 tips and 1109 internal nodes.

Tip labels:

OTU1144, OTU9, OTU741, OTU254, OTU15, OTU719, ...

Node labels:

n1, n2, n3, n4, n5, n6, ...

Rooted; includes branch lengths.

#Remove low abundance taxa, add pseudocount of 0.001 throughout

> filter_taxa (snx1, function(x) sum(x > 3) > (0.2*length(x)), TRUE)

> snx1<- filter_taxa(snx, function(x) sd(x)/mean(x) > 3.0, TRUE)

> snx1 <- transform_sample_counts(snx1, function(x) x+0.001)

#Extract components for philr log-transformations

> otu.table <- out_table(snx1)

> tree <- phy_tree(snx1)

#Perform isometric log-ratio transformation using philr

> snx.philr <-philr(otu.table, tree, part.weights='uniform', ilr.weights='uniform')

#Ordinate in philr space; extract PCoA scores to be plotted in GraphPad Prism

> snx.dist <- dist(snx.philr, method="euclidean")

> snx.pcoa <- ordinate(snx1, 'PCoA', distance=snx.dist)

> vectors<-snx.pcoa$vectors

> write.csv(vectors,"vectors.csv")

#Perform ADONIS using Euclidean distances in philr space, by both batch and treatment class:

> metadata <- as(sample_data(snx1), "data.frame")

> adonis2(snx.dist ~ Batch, data = metadata)

Permutation test for adonis under reduced model

Terms added sequentially (first to last)

Permutation: free

Number of permutations: 999

adonis2(formula = snx.dist ~ Batch, data = metadata)

Df SumOfSqs R2 F Pr(>F)

Batch 1 30353 0.09725 2.37 0.001 ***

Residual 22 281762 0.90275

Total 23 312116 1.00000

---

Signif. codes: 0 ‘***’ 0.001 ‘**’ 0.01 ‘*’ 0.05 ‘.’ 0.1 ‘ ’ 1

> adonis2(snx.dist ~ Treatment, data = metadata)

Permutation test for adonis under reduced model

Terms added sequentially (first to last)

Permutation: free

Number of permutations: 999

adonis2(formula = snx.dist ~ Treatment, data = metadata)

Df SumOfSqs R2 F Pr(>F)

Treatment 1 15039 0.04818 1.1137 0.227

Residual 22 297076 0.95182

Total 23 312116 1.00000

#Perform OPLS-DA for both batch and treatment class

> oplsCh <-opls(otu, sample_data(snx1)$Batch, predI=1, orthoI=2)

OPLS-DA

24 samples x 1110 variables and 1 response

standard scaling of predictors and response(s)

R2X(cum) R2Y(cum) Q^2^Y(cum) RMSEE pre ort pR2Y pQ2

Total 0.251 0.986 0.573 0.0634 1 2 0.8 (<)0.05

> oplsTx <-opls(otu, sample_data(snx)$Treatment, predI=1, orthoI=2)

24 samples x 1110 variables and 1 response

standard scaling of predictors and response(s)

R2X(cum) R2Y(cum) Q^2^Y(cum) RMSEE pre ort pR2Y pQ2

Total 0.216 0.993 0.206 0.0466 1 2 0.15 0.2

#Estimate alpha diversity, export sample values for plotting and significance testing in GraphPad Prism

> alpha<-estimate_richness(snx1, measures=c("InvSimpson", "Shannon"))

> write.csv(alpha, "philralpha.csv")

#Prepare data for ANCOM analysis:

> Sample.ID <- colnames(otu.table)

> otu <- t(otu.table)

> otu <- cbind(Sample.ID, otu)

> meta <- sample_data(snx1)

> meta <- cbind(Sample.ID, otu.table)

Use ANCOM to estimate differentially abundant OTUs by batch:

#ANCOM code for functions Ancom.w and ANCOM.main manually entered after download from https://sites.google.com/site/siddharthamandal1985/research.

>comparison_test=ANCOM.main(OTUdat=otu,Vardat=meta,adjusted=FALSE,repeated=F,main.var="Batch", multcorr=2, sig=0.05,prev.cut=0.90)

> comparison_test$W.taxa

> bybatch<-comparison_test$W.taxa

otu.names W_stat detected_0.9 detected_0.8 detected_0.7 detected_0.6

OTU415 1080 TRUE TRUE TRUE TRUE

OTU296 1059 TRUE TRUE TRUE TRUE

OTU162 1017 TRUE TRUE TRUE TRUE

OTU274 1010 TRUE TRUE TRUE TRUE

OTU54 999 TRUE TRUE TRUE TRUE

OTU747 989 FALSE TRUE TRUE TRUE

OTU287 978 FALSE TRUE TRUE TRUE

OTU17 977 FALSE TRUE TRUE TRUE

OTU377 954 FALSE TRUE TRUE TRUE

OTU303 947 FALSE TRUE TRUE TRUE

OTU991 946 FALSE TRUE TRUE TRUE

OTU345 925 FALSE TRUE TRUE TRUE

OTU554 924 FALSE TRUE TRUE TRUE

OTU221 920 FALSE TRUE TRUE TRUE

OTU1016 903 FALSE TRUE TRUE TRUE

OTU36 898 FALSE TRUE TRUE TRUE

OTU536 884 FALSE FALSE TRUE TRUE

OTU489 870 FALSE FALSE TRUE TRUE

OTU632 860 FALSE FALSE TRUE TRUE

OTU375 847 FALSE FALSE TRUE TRUE

OTU922 845 FALSE FALSE TRUE TRUE

OTU280 845 FALSE FALSE TRUE TRUE

OTU158 813 FALSE FALSE TRUE TRUE

OTU121 809 FALSE FALSE TRUE TRUE

OTU845 799 FALSE FALSE TRUE TRUE

OTU455 798 FALSE FALSE TRUE TRUE

OTU737 779 FALSE FALSE TRUE TRUE

OTU168 769 FALSE FALSE FALSE TRUE

OTU562 749 FALSE FALSE FALSE TRUE

OTU218 719 FALSE FALSE FALSE TRUE

OTU401 714 FALSE FALSE FALSE TRUE

OTU248 707 FALSE FALSE FALSE TRUE

OTU496 673 FALSE FALSE FALSE TRUE

OTU629 664 FALSE FALSE FALSE FALSE

OTU367 655 FALSE FALSE FALSE FALSE

OTU214 642 FALSE FALSE FALSE FALSE

>comparison_test=ANCOM.main(OTUdat=otu,Vardat=meta,adjusted=FALSE,repeated=F,main.var="Treatment", multcorr=2, sig=0.05,prev.cut=0.90)

> comparison_test$W.taxa

> bytreatment<-comparison_test$W.taxa

otu.names W_stat detected_0.9 detected_0.8 detected_0.7 detected_0.6

OTU33 1042 TRUE TRUE TRUE TRUE

OTU979 982 FALSE TRUE TRUE TRUE

OTU288 957 FALSE TRUE TRUE TRUE

OTU503 906 FALSE TRUE TRUE TRUE

OTU329 858 FALSE FALSE TRUE TRUE

OTU2 856 FALSE FALSE TRUE TRUE

50 602 FALSE FALSE FALSE FALSE

391 533 FALSE FALSE FALSE FALSE

1080 431 FALSE FALSE FALSE FALSE

#Get total abundances of these differentially expressed OTUs

> otu<-otu_table(snx)

>otu<-(otu-0.001)

>rowsums<-rowSums(otu) #Total abundances for each OTU

>sum(rowsums)

4209304 #Total abundances of all OTUs in all samples

> batchotus<-c(415,296,162,274,54,747,287,17,377,303,991,345,554,221,1016,36,536,489,632,375,922,280,158,121,845,455,737,168,562,218,401,248,496)

> batchnumbers<-subset(rowsums,rowsums$rownames.otu %in% batchotus)

> sum(treatmentnumbers)

160010 #Total abundances of all OTUs showing differences between batches

> 160010/4209304

0.038 #Proportional abundance of all OTUs showing differences between batches

> treatmentotus<- c(33,979,288,503,329,2)

> treatmentnumbers<-subset(rowsums,rowsums$rownames.otu %in% treatmentotus)

> sum(treatmentnumbers)

216027 #Total abundances of all OTUs showing differences between treatment classes

> 216027/4209304

0.051 #Proportional abundance of all OTUs showing differences between treatment classes

#Agglomeration of taxa to allow phylum level analysis

> phylum<-tax_glom(snx, taxrank=”Rank2”)

> otu1<-t(otu.table(phylum))

> meta1<-sample_data(phylum)

#Repeated for other taxonomic ranks to allow the different analyses below

#Phylum level analysis by batch

>comparison_test=ANCOM.main(OTUdat=otu1,Vardat=meta1,adjusted=FALSE,repeated=F,main.var="Batch", multcorr=2, sig=0.05,prev.cut=0.90)

> comparison_test$W.taxa

otu.names W_stat detected_0.9 detected_0.8 detected_0.7 detected_0.6

OTU0 0 FALSE FALSE FALSE FALSE

OTU1 0 FALSE FALSE FALSE FALSE

OTU10 0 FALSE FALSE FALSE FALSE

#Phylum level analysis by treatment

>comparison_test=ANCOM.main(OTUdat=otu1,Vardat=meta1,adjusted=FALSE,repeated=F,main.var="Treatment", multcorr=2, sig=0.05,prev.cut=0.90)

> comparison_test$W.taxa

otu.names W_stat detected_0.9 detected_0.8 detected_0.7 detected_0.6

OTU0 0 FALSE FALSE FALSE FALSE

OTU1 0 FALSE FALSE FALSE FALSE

OTU10 0 FALSE FALSE FALSE FALSE

#Order level analysis by batch

otu.names W_stat detected_0.9 detected_0.8 detected_0.7 detected_0.6

OTU345 6 FALSE FALSE FALSE FALSE

OTU211 6 FALSE FALSE FALSE FALSE

OTU475 1 FALSE FALSE FALSE FALSE

#Order level analysis by treatment

otu.names W_stat detected_0.9 detected_0.8 detected_0.7 detected_0.6

OTU0 0 FALSE FALSE FALSE FALSE

OTU345 0 FALSE FALSE FALSE FALSE

OTU1 0 FALSE FALSE FALSE FALSE

#Class level analysis by batch

otu.names W_stat detected_0.9 detected_0.8 detected_0.7 detected_0.6

OTU562 17 FALSE FALSE TRUE TRUE

OTU629 17 FALSE FALSE TRUE TRUE

OTU345 11 FALSE FALSE FALSE FALSE

OTU211 11 FALSE FALSE FALSE FALSE

OTU933 3 FALSE FALSE FALSE FALSE

#OTU562 from class *Pseudomonadales*

#OTU629 from class *Thermoanaerobacteraeles*

#Class level analysis by treatment

otu.names W_stat detected_0.9 detected_0.8 detected_0.7 detected_0.6

OTU103 4 FALSE FALSE FALSE FALSE

OTU933 2 FALSE FALSE FALSE FALSE

OTU579 1 FALSE FALSE FALSE FALSE

#Family level analysis by batch

otu.names W_stat detected_0.9 detected_0.8 detected_0.7 detected_0.6

OTU629 33 FALSE FALSE TRUE TRUE

OTU345 24 FALSE FALSE FALSE FALSE

OTU562 18 FALSE FALSE FALSE FALSE

#OTU629 from class *Thermoanaerobacteraceae*

#Family level analysis by treatment

otu.names W_stat detected_0.9 detected_0.8 detected_0.7 detected_0.6

OTU475 3 FALSE FALSE FALSE FALSE

OTU103 1 FALSE FALSE FALSE FALSE

OTU579 1 FALSE FALSE FALSE FALSE

#Genus level analysis by batch

otu.names W_stat detected_0.9 detected_0.8 detected_0.7 detected_0.6

OTU629 99 FALSE FALSE TRUE TRUE

OTU345 70 FALSE FALSE FALSE FALSE

OTU241 67 FALSE FALSE FALSE FALSE

#Genus level analysis by treatment

otu.names W_stat detected_0.9 detected_0.8 detected_0.7 detected_0.6

OTU0 0 FALSE FALSE FALSE FALSE

OTU348 0 FALSE FALSE FALSE FALSE

OTU719 0 FALSE FALSE FALSE FALSE
